# Impact of Breastfeeding Practices on Autistic Traits in Chinese Children Aged from 3 to 4 Years: Cross-Sectional Study

**DOI:** 10.3390/nu17050836

**Published:** 2025-02-28

**Authors:** Jianhui Yang, Lu Gao, Esben Strodl, Jieping Chen, Feng Tong, Weiqing Chen

**Affiliations:** 1 Ningbo Municipal Centre for Disease Control and Prevention, Ningbo 315010, China; yangjh83@mail3.sysu.edu.cn (J.Y.);; 2 Hangzhou Lin’an District Centre for Disease Control and Prevention (Hangzhou Lin’an District Health Supervision Institute), Hangzhou 311399, China; 3 School of Psychology and Counselling, Queensland University of Technology, Brisbane, QLD 4059, Australia; e.strodl@qut.edu.au; 4 Department of Epidemiology, School of Public Health, Sun Yat-sen University, Guangzhou 510080, China; 5 Department of Information Management, Xinhua College, Sun Yat-sen University, Guangzhou 510080, China

**Keywords:** exclusive breastfeeding duration, breastfeeding duration, autistic traits, children, China

## Abstract

**Background**: Although breastfeeding has been extensively documented to confer health benefits to infants in the early stages of their lives, the sustained influence that it has on autistic traits throughout childhood remains unclear. This study endeavors to explore the correlation between the length of exclusive breastfeeding, the overall duration of breastfeeding, and the manifestation of autistic traits in Chinese children. **Methods**: A cross-sectional study was conducted among 17,382 three-year-olds residing in Longhua District, Shenzhen, China. The participants’ socio-economic status, breastfeeding patterns, and autistic traits were recorded using questionnaires. Breastfeeding durations were analyzed both as continuous and categorical variables. **Results**: Of the 17,382 children studied, 666 exhibited autistic traits. Exclusive breastfeeding for ≥2 months was notably associated with a decreased risk of developing autistic traits when it was assessed using continuous measures. Furthermore, children who were breastfed for 13 months or longer exhibited a lower risk of developing autistic traits, as compared to those who were breastfed for 6 months or less, when this was measured using categorical methods. Similarly, when it was assessed using continuous measures, children who were breastfed for at least 8 months also demonstrated a reduced risk of developing autistic traits. Linear relationships were discernible between exclusive breastfeeding duration, overall breastfeeding duration, and autistic traits. **Conclusions**: Exclusive breastfeeding for a period from 2 to 6 months, coupled with extended breastfeeding lasting for at least 8 months, demonstrated a beneficial effect in alleviating autistic traits among Chinese children. These findings contribute to refining and strengthening the existing recommendations concerning breastfeeding practices.

## 1. Introduction

Autism Spectrum Disorder (ASD) is a developmental condition distinguished by impairments in social and communicative interactions, accompanied by narrow and repetitive patterns of interests, behaviors, or activities, as outlined by the American Psychiatric Association [1]. The prevalence of ASD is estimated to be about 2.3% in the United States and 0.7% in China [2,3], but some researchers believe that it is now on the rise [4]. Research suggests that ASD traits exist on a spectrum within the general population, with clinical ASD representing the most severe manifestations of this spectrum [5,6].

Individuals diagnosed with ASD face numerous cognitive and behavioral challenges, including difficulties with social skills, attention shifting, communication, imagination, and detail-oriented focus [7,8,9]. Notably, these traits can also be observed, albeit to a lesser extent, in individuals who do not meet the clinical criteria for an ASD diagnosis [10,11,12]. These subclinical autistic traits, defined as deficits in socialization, communication, and repetitive behaviors that fall short of the formal diagnostic criteria for ASD [5], have garnered significant attention in recent years.

Importantly, studies have shown that the genetic and environmental influences on these subclinical traits are consistent across the entire range of impairment within the continuous autistic trait spectrum [13]. This indicates an etiologic overlap between extreme scores, mild impairment, and subthreshold autism-like behavior. By investigating the modifiable factors linked to autism-like traits during early life stages, researchers can gain an invaluable understanding of the core mechanisms of clinical ASD. Such insights pave the way for potential early interventions, offering opportunities to mitigate the behavioral and emotional challenges associated with the condition.

The breastfeeding practices of infants may be among the potentially modifiable factors influencing the manifestation of autistic traits. The American Academy of Pediatrics (AAP), the United Nations International Children’s Emergency Fund (UNICEF), and the World Health Organization (WHO) are among the numerous global organizations that advocate for exclusive breastfeeding for the first six months of an infant’s life, followed by the introduction of suitable complementary foods and continued breastfeeding until at least the age of two, and sometimes beyond [14,15,16]. Breastfeeding is a complex and interactive social mother–child behavior that goes beyond mere nourishment. It offers numerous benefits to the child, including providing essential nutrients for physical and neurologic development, bolstering the immune system to protect against infections, and reducing the risk of food allergies, asthma, and cardiovascular diseases [17,18]. These advantages underscore the importance of breastfeeding in fostering the overall health and well-being of infants. Globally, it was estimated by the World Health Organization (WHO) [19] that only 44% of infants aged 0–6 months are exclusively breastfed. However in China, this figure dropped even further, with the exclusive breastfeeding rate within the first six months being observed to be less than 30% [20]. Similarly, the percentage of infants who continued to receive breastmilk for a duration exceeding twelve months was also notably low, amounting to only 18% [21]. These less than optimal rates of exclusive breastfeeding, especially in China, highlight this parental feeding behavior as a potentially significant risk factor for chronic disease, ill health, and neurodevelopmental delay in infants.

To date, studies evaluating the association between breastfeeding practices and child ASD or autistic traits have reported conflicting results [22]. Numerous studies have revealed that children with ASD are less likely to be exclusively breastfed during the first six months of life and tend to have a shorter duration of breastfeeding compared to their non-ASD peers [23,24,25,26,27,28]. These findings demonstrate the potential protective role that breastfeeding may play in children’s neurodevelopment. For example, a retrospective case–control study conducted in Israel revealed a reduced likelihood of an ASD diagnosis in children associated with exclusive breastfeeding in the first six months and a longer breastfeeding duration [28]. In contrast, other studies have found no associations between ASD and exclusive breastfeeding or breastfeeding duration [29,30,31,32,33]. For example, in a population cohort study involving 191,745 Scottish school children, Adams and Pell found no association between exclusive breastfeeding and the rate of ASD [29]. Similarly, a large nationally representative survey conducted with US children found that a diagnosis of ASD was unassociated with breastfeeding history [30]. Furthermore, a systematic review and meta-analysis conducted by Gozy and his colleagues found that there was no significant association between patterns of breastfeeding and the risk of ASD among offspring [33].

However, previous studies have primarily concentrated on either exclusive breastfeeding duration or breastfeeding duration in isolation, overlooking the distinct effects that these two breastfeeding modes may have on ASD in children [32,34]. Moreover, past research has predominantly analyzed exclusive breastfeeding duration and overall breastfeeding duration as categorical variables, leaving the continuous dose–response relationship between these durations and the manifestation of autistic traits in children unclear. The mixed findings to date necessitate further exploration through large-scale population studies using both categorical and continuous measures for both exclusive and overall breastfeeding duration. Additionally, the bulk of these studies have been confined to Western populations [35], resulting in a significant lack of data for Asian populations, especially Chinese children.

Therefore, in our study, we utilized data from the Longhua Child Cohort Study (LCCS) to investigate the link between the duration of exclusive breastfeeding, overall breastfeeding duration, and autistic traits among Chinese children. Our focus was to delineate the optimal breastfeeding durations that are particularly relevant for Chinese populations, considering the AAP, UNICEF, and WHO recommendations. By doing so, we aim to add to the evidence base guiding breastfeeding practices associated with the prevention and management of autistic traits in children.

## 2. Materials and Methods

### 2.1. Study Population

The LCCS, a meticulously designed and ongoing prospective cohort study, is dedicated to examining the intricate influences of early-life family and school environments on neurobehavioral development in children residing in the Longhua District of Shenzhen, China. With a particular focus on hyperactivity, conduct problems, and autistic traits [36], the LCCS has been continuously implemented since its inception in September 2014.

Adopting a standardized protocol, the study annually enrolls child–mother pairs from all preschools within the Longhua District. This protocol involves preschool educators inviting mothers to participate by signing an informed consent form and completing a structured questionnaire administered by the mother. The exclusion criteria are children with known severe physical or mental conditions. In 2017, a total of 17,867 child–mother pairs, with children aged from 3 to 4 years, were enrolled in the baseline surveys of the LCCS. After excluding children’s mothers who did not provide complete information on the questionnaire (*n* = 168) and children who were not born as singletons (*n* = 485), data from 17,382 (97.29%) child–mother dyads were included in the analysis.

The LCCS received ethical clearance from the Human Research Ethics Committee of the School of Public Health, Sun Yat-sen University, Guangzhou, China (Approval Number: 2015–016, date of approval: 4 May 2015). Throughout the study, stringent adherence to the ethical principles outlined in the Declaration of Helsinki has been ensured.

Data collection within the LCCS has been comprehensive and detailed. The socio-demographic attributes of both children and their parents, including age, gender, marital status, educational attainment, household income, and occupation, have been meticulously documented. Parents also provide self-reported information on the duration of exclusive breastfeeding and overall breastfeeding that their children received. Furthermore, the parents rate their children’s exhibition of autistic traits.

### 2.2. Outcome Definition

In this study, the Autism Behavior Checklist (ABC) was employed to assess the presence of autistic traits in children. Since its inception in 1980, this tool has gained widespread international recognition for screening Autism Spectrum Disorder (ASD) symptoms in individuals aged between 18 months and 35 years [37]. Introduced to China by Yang and colleagues, the ABC has demonstrated a robust reliability and validity [38]. The ABC is a 57-item screening checklist for autism containing five sub-scales (body behavior, sensory, self-care, language, and social interaction). It is designed for parent interviews [39]. Each item is rated on a scale from 1 to 4, with the total ABC score ranging from 0 to 158, calculated by summing the scores of all items. A higher score indicates a greater presence of autism-like behaviors. The scale’s developer recommended using cut-off values of 53 and 68 for screening and diagnosis, respectively [37]. However, in China, a score of 31 has consistently been identified as the optimal cut-off for screening children with autism-like behaviors [40,41,42,43]. Therefore, based on this previous evidence, a cut-off value of 31 was adopted in this study as the threshold for identifying autism-like behaviors.

### 2.3. Measurements of Exclusive Breastfeeding Duration and Breastfeeding Duration

Exclusive breastfeeding duration refers to the time span during which an infant receives solely breastmilk, without any supplementary liquids or solid foods. The overall duration of breastfeeding spans the entire period that a mother nurses her child, either exclusively with her own milk or combined with complementary foods, until weaning occurs [16]. In our study, mothers were prompted to self-report their breastfeeding durations by answering two tailored questions on our questionnaire. One question asked for the total duration of exclusive breastfeeding in months, while the other inquired about the overall duration of breastfeeding in months.

### 2.4. Covariates

Demographic information and other self-reported risk factors were collected via questionnaires, including the child’s gender, whether the child is an only child, the parents’ marital status, education level, family income, parental age at the child’s birth, and gestational diseases, as well as the timing of introducing complementary food. We also collected information on mother-reported birth weight and preterm birth.

### 2.5. Covariates Adjustment

First, we chose the potential covariates (child’s gender, age, birth weight, preterm birth, single child or not, parents’ marital status, education level, family income, parity, parental age at the time of the child’s birth, and gestational diseases) based on previous relevant publications [44,45,46,47,48]. Second, we put all these potential confounders into a multiple logistic model with the presence of autism-like behaviors as the dependent variable. If a certain potential confounder had a *p* value that was equal to or over 0.1 (*p* ≥ 0.1), then this variable was excluded from the list of potential covariates. We then repeated the process again until the *p* vales of all the covariates included in the multiple logistic models were less than 0.1 (*p* < 0.1). As a result of this process, we determined that the child’s gender, age, parental age at the time of the child’s, parental education level birth, the parents’ marital status, family income, parity, and gestational diseases should be the covariates included in the final analysis.

### 2.6. Statistical Analysis

Continuous variables are presented either as the mean ± standard deviation (SD) or as the median with the interquartile range (IQR), whereas categorical variables are reported using frequencies and percentages. Depending on the data distribution, baseline continuous variables were analyzed using the *t*-test, Mann–Whitney U-test, one-way analysis of variance (ANOVA), or Kruskal–Wallis test. For categorical variables, the chi-square test or Fisher’s exact probability method were employed.

For statistical analysis, exclusive breastfeeding was treated in the following two ways: as a categorical variable, indicating whether the child was exclusively breastfed for the first six months of life, and as a continuous variable, reflecting the exact duration of exclusive breastfeeding. Likewise, the overall breastfeeding duration was analyzed both categorically (classified into ≤6 months, 7 to 12 months, or ≥13 months) and continuously. This dual perspective allowed for a detailed and nuanced examination of the data, as further elaborated in the subsequent statistical analysis section.

First, we examined differences in the baseline data between the autistic group and the non-autistic group. Second, drawing upon previous articles [26,28,33,49], we categorized the duration of exclusive breastfeeding during the first six months into the following two distinct patterns: exclusive breastfeeding throughout the first six months of life and non-exclusive breastfeeding within the same period. Based on previous studies [28,50], we segmented the overall breastfeeding duration into the following three phases: ≤6 months, 7–12 months, and ≥13 months. The associations between exclusive breastfeeding, breastfeeding, and autism-like behaviors in offspring were examined using logistic regression models. We initially established crude models and subsequently adjusted for the child’s gender, age, parental age at the time of the child’s, parental education level birth, the parents’ marital status, family income, parity, and gestational diseases. The results are presented as crude odds ratios (cOR) and adjusted odds ratios (aOR), both with their respective 95% confidence intervals (95% CIs). Additionally, the relationships between these breastfeeding practices and the children’s ABC scores were investigated using linear regression models, with similar adjustments for the aforementioned variables. The outcomes are reported as crude Beta coefficients (cβ) and adjusted Beta coefficients (aβ), again accompanied by their 95% CIs. Third, restrictive cubic splines were used to examine the shape of the relationship of exclusive breastfeeding duration and overall breastfeeding duration with autistic traits in children after controlling for the aforementioned variables. In addition, we performed a subgroup analysis stratified by the children’s sex and other variables. All analyses were performed in R language, and *p* values of <0.05 on both sides were considered statistically significant.

## 3. Results

### 3.1. Demographic Characteristics

A total of 17,382 preschoolers, specifically aged from 3 to 4 years, participated in the survey with their parents (Table 1). The autistic traits group comprised 666 children, consisting of 422 boys and 244 girls. The mean birth weight for this group was 3428.42 g (SD = 927.33), and the mean birth length was 50.24 cm (SD = 5.70). In contrast, the non-autistic group consisted of 16,716 children, including 8991 boys and 7725 girls. The mean birth weight for this larger group was 3419.01 g (SD = 851.91), and the mean birth length was 51.04 cm (SD = 5.49). The two groups exhibited statistically significant differences in various demographic and health-related factors, including gender, birth length, parents’ age at childbirth, parents’ education level, family income, marital status, whether the child was an only child, threatened abortion, gestational hypertension, and preeclampsia/eclampsia (*p* < 0.05). Therefore, we corrected for these demographic factors in multivariate logistic regression analysis and linear regression analysis.

Notably, these two groups also exhibited statistically significant disparities in the duration of exclusive breastfeeding and overall duration of breastfeeding. Specifically, in the non-autistic trait group, the average duration of exclusive breastfeeding was 3.35 months (SD = 3.16), whereas in the autistic traits group, it was notably shorter, averaging at just 3.02 months (SD = 3.02). Similarly, regarding the duration of breastfeeding, the non-autistic group were breastfed for an average of 8.76 months (SD = 5.75) compared to the autistic traits group, which had an average breastfeeding duration of only 8.31 months (SD = 5.57) (See more details in Table 1).

### 3.2. Differences in Autistic Traits in Children with Different Breastfeeding Patterns

Table 2 presents a comparison of autistic traits in children based on their exclusive breastfeeding status during the first six months of life. Our findings revealed that children who were exclusively breastfed in this period exhibited a lower prevalence of autistic traits (4.00% versus 3.30%, *p* = 0.030) compared to those who were not. Furthermore, these exclusively breastfed children scored lower across all sub-scales of the ABC, with the exception of the body and object use scores. Specifically, the overall ABC scores were 6.21 versus 5.55 (*p* = 0.001), the sensory scores were 0.85 versus 0.74 (*p* = 0.009), the relating scores were 2.02 versus 1.88 (*p* = 0.046), the language scores were 1.01 versus 0.85 (*p* = 0.001), and the social and self-help scores were 1.72 versus 1.55 (*p* = 0.001) (see more details in Table 2).

Additionally, as the overall duration of breastfeeding increased, there was a notable decrease in the prevalence of autistic traits among children (*p* for trend < 0.05). Furthermore, these children exhibited lower scores on all scales of the ABC (*p* for trend < 0.05) (for further details, please refer to Table 3).

### 3.3. Relationship Between Exclusive Breastfeeding Duration and Autistic Traits in Children

Table 4 displays the association between the categorical measure of exclusive breastfeeding in the first six months of life and children’s autistic traits. After accounting for potential confounders, the result indicates no statistically significant relationship between the duration of exclusive breastfeeding and degree of autistic traits in young children.

Table 5 presents the associations between the categorical measure of exclusive breastfeeding in the first six months of life and the continuous measures of the children’s ABC scores, as well as the sub-scale scores of the ABC questionnaire. Using multivariable linear regression models and accounting for potential confounders, the findings indicate that children who were exclusively breastfed during this initial period exhibited significantly lower ABC scores, including lower scores in the language, social, and self-help domains when compared to their counterparts who were not exclusively breastfed.

**Figure 1:**
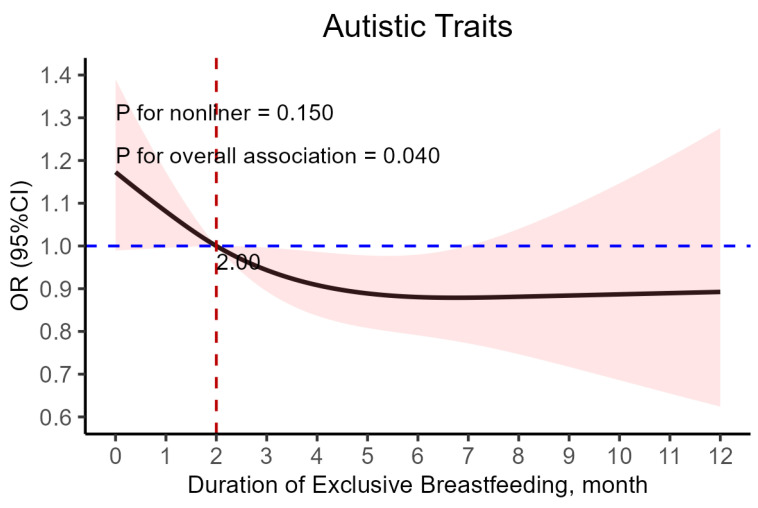
The full adjusted relationship between duration of exclusive breastfeeding and children’s autistic traits ^†^. ^†^ Adjusted for child’s gender, birth weight (g), birth length (cm), preterm birth status, whether the child was an only child, parents’ age at child’s birth, parents’ marital status, parents’ education level, family income, supplementation time (month), threatened abortion, and gestational diseases (gestational hypertension and preeclampsia/eclampsia).

Moreover, a significant linear relationship was observed between the duration of exclusive breastfeeding and the risk of autistic traits in children, while the nonlinear regression model was not significant. Specifically, the likelihood of autistic traits in a child decreased from an odds ratio of close to 1.2 to 1.0 when the duration of exclusive breastfeeding increased from 0 months to 2 months (Figure 1). Given the heightened probability of autistic traits among children with less than 2 months of exclusive breastfeeding, a logistic regression model was developed, using <2 months of exclusive breastfeeding as the reference group (Table 6). Notably, children who were exclusively breastfed for 2 months or more were found to be less likely to develop autistic traits compared to those who were exclusively breastfed for less than 2 months, with an aOR of 0.848 (95% CI: 0.725–0.993, *p* = 0.0404).

### 3.4. Relationship Between Overall Breastfeeding Duration and Autistic Traits in Children

Table 7 exhibits the relationship between the overall duration of breastfeeding and the presence of autistic traits in children. After adjusting for potential confounders, the results revealed a significant decrease in the risk of autistic traits among children who were breastfed for 13 months or more when compared to those who were breastfed for 6 months or less. Specifically, the aOR for developing autistic traits was 0.709 (95% CI: 0.561–0.897, *p* = 0.0041) for children with ≥13 months of overall breastfeeding time, indicating a protective effect against the development of autistic traits.

Table 8 displays the multiple linear regression models between the overall duration of breastfeeding and children’s scores on the ABC questionnaire, including its sub-scale scores, after adjusting for covariates. The results revealed that children who were breastfed for durations of 7–12 months or 13 months and beyond exhibited significantly lower ABC scores, as well as lower scores on all sub-scales of the ABC questionnaire, except for the relating sub-scale, compared to those who were breastfed for less than 6 months.

**Figure 2:**
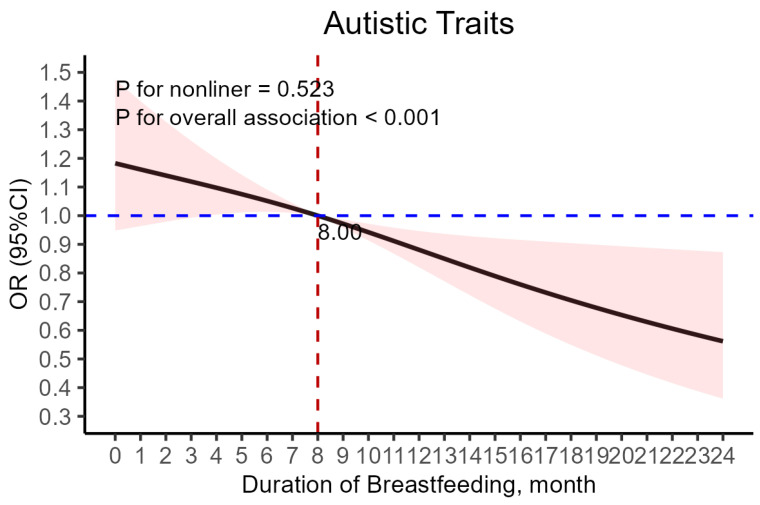
The full adjusted relationship between duration of breastfeeding and children’s autistic traits ^†^. ^†^ Adjusted for child’s gender, birth weight (g), birth length (cm), preterm birth status, whether the child was an only child, parents’ age at child’s birth, parents’ marital status, parents’ education level, family income, supplementation time (month), threatened abortion, and gestational diseases (gestational hypertension and preeclampsia/eclampsia).

A statistically significant linear relationship was established between the overall duration of breastfeeding and the risk of autistic traits in children, while the nonlinear model was not significant. Furthermore, an inflection point was identified, as illustrated in Figure 2, suggesting that the odds ratio of a child having autistic traits dropped below 1.0 when a child was breastfed for ≥8 months. Given the elevated risk of autistic traits observed in children who were breastfed for less than 8 months, a logistic regression model was formulated, with breastfeeding duration of <8 months serving as the baseline reference group (see Table 6). Notably, the results indicated that children who were breastfed for 8 months or longer had a lower likelihood of developing autistic traits compared to those breastfed for less than 8 months. Specifically, the aOR was 0.798 (95% CI: 0.682–0.934; *p* = 0.0050).

### 3.5. Subgroup Analysis

We conducted stratified analyses to examine whether a range of demographic variables moderated the relationships between exclusive breastfeeding during the first six months of life (yes vs. no) and the overall duration of breastfeeding (categorized into ≤6 months, 7–12 months, and ≥13 months) on the odds of a child experiencing autistic traits. The results indicated that none of the evaluated variables significantly altered this association. These variables encompassed gender (boys vs. girls; *p*-interaction = 0.565), maternal age at childbirth (≤35 years vs. >35 years; *p*-interaction = 0.468), paternal age at childbirth (≤40 years vs. >40 years; *p*-interaction = 0.805), maternal education level (junior high school or lower, high school, and college or higher; *p*-interaction = 0.964), paternal education level (junior high school or lower, high school, and college or higher; *p*-interaction = 0.931), family income (<5000, 5001–10,000, 10,001–20,000, and >20,000; *p*-interaction = 0.870), marital status (married vs. single; *p*-interaction = 0.246), whether the child was an only child (no vs. yes; *p*-interaction = 0.131), a history of threatened abortion (no vs. yes; *p*-interaction = 0.620), the presence of gestational hypertension (no vs. yes; *p*-interaction = 0.815), preeclampsia/eclampsia (no vs. yes; *p*-interaction = 0.952), and the introduction of complementary foods at six months (no vs. yes; *p*-interaction = 0.988). Furthermore, we discovered notable interactions between family income and the overall duration of breastfeeding in relation to children’s autistic traits (*p*-interaction = 0.008), as well as between gestational hypertension and the overall breastfeeding period in connection with these traits (*p*-interaction = 0.049). Conversely, no statistically significant interactions were identified between the overall breastfeeding duration or any other stratifying variables and the risk of autistic traits in children (see more details in Table 9).

Upon conducting a series of subgroup analyses stratified by the children’s sex, we observed notable associations between the duration of exclusive breastfeeding and overall breastfeeding duration with autistic traits, persisting predominantly in boys. Conversely, these associations were not pronounced in girls. Specifically, in boys who were exclusively breastfed in the first six months, compared to those who were not, we noted lower ABC scores across nearly all sub-scales, with the exception of their body and object use scores and language scores (Table S2). Comparable results were obtained when utilizing multiple linear models (Table S3).

Regarding the overall duration of breastfeeding, when using a categorical measure of the presence of autistic traits, boys who were breastfed for 7–12 months or for 13 months or more exhibited a reduced risk of autistic traits, compared to those who were breastfed for six months or less (Table S4). These associations were not significant for girls. Furthermore, these boys also demonstrated lower ABC scores across almost all sub-scales, excluding their relating scores (Table S5). Notably, the duration of breastfeeding maintained a significant association with ABC scores and all sub-scales in both comparison groups (Table S6).

## 4. Discussion

In this cross-sectional study, we successfully enrolled a total of 17,382 children from China. Among this population, 666 children exhibited autistic traits. After adjusting for various covariates, we found an independent and inverse association between exclusive breastfeeding, as well as overall breastfeeding, and the presence of autistic traits. Furthermore, our findings unveiled linear associations between the duration of exclusive breastfeeding, as well as the duration of overall breastfeeding, and the manifestation of autistic traits in children. Specifically, children who were exclusively breastfed for a longer duration (≥2 months) and breastfed for an extended period (≥8 months) exhibited a decreased risk of developing autistic traits, in contrast to those who underwent shorter durations of exclusive breastfeeding (<2 months) and overall breastfeeding (<8 months). Finally, numerous subgroup analyses revealed that these beneficial associations were more pronounced in boys compared to girls. Therefore, these findings imply that optimizing the duration of exclusive breastfeeding and prolonging the overall duration of breastfeeding may have positive implications for the neurodevelopment of children.

These results are an important contribution to the literature, given that the evidence linking exclusive breastfeeding to autistic traits in children has been characterized by limited and inconsistent findings. A national population cohort study involving 191,745 Scottish schoolchildren found no association between exclusive breastfeeding in the first 6 to 8 weeks and the risk of autism in these children (exclusive 0.88 [0.77,1.01], *p* = 0.074 and mixed 1.01 [0.84,1.22], *p* = 0.903) [29]. Additionally, a meta-analysis revealed a nonsignificant reduction in the risk of ASD with exclusive breastfeeding (pooled OR = 0.952, 95%CI = 0.826–1.096, *p* = 0.169) [33]. However, several studies have indicated a negative association between exclusive breastfeeding and the risk of autistic traits in children [23,24,28,34,49,51]. For instance, a retrospective cohort study conducted in South Korea with 188,052 children found that exclusive breastfeeding in the first 4 to 6 months significantly decreased the risk of ASD in children at two years old (RR = 0.72, 95% CI = 0.57–0.89) [49]. These inconsistent findings may be partially attributed to various factors, including differences in study areas (predominantly Western countries), varied definitions of outcomes (such as parent reports [51], DSM-5 criteria [34], or screening only [29]), varying study sample sizes, and different confounding factors in these studies. However, while previous studies have predominantly examined the relationship between breastfeeding duration and autism using categorical variables, our study significantly contributes to the scientific literature by employing both categorical and continuous measures for both independent and dependent variables. Our findings align with the majority of research outlined in the aforementioned literature, suggesting that exclusive breastfeeding decreases the risk of children developing autistic traits. Furthermore, we discovered that the risk of autistic traits in children diminishes when the duration of exclusive breastfeeding is at least 2 months. This risk continues to decrease as the duration of exclusive breastfeeding increases, reaching a stabilizing protective effect at around 6 months of exclusive breastfeeding. However, it is crucial to emphasize that additional prospective cohort studies are required to definitively confirm our observations.

Our investigation further examined the relationship between total breastfeeding duration and the manifestation of autistic traits in children. Our study found that children with breastfeeding duration of ≥13 months had a lower risk of developing autistic traits. Similar associations were also found in other studies [26,27]. For instance, a community-based case–control study at six sites in the Unites States found that children with ASD were less likely to report a duration of breastfeeding in the high (≥12 months) versus low tertile (<6 months) (aOR = 0.61, 95%CI = 0.45–0.84) [26]. Compared with this study, we revealed a significant negative association, with infants breastfed for ≥8 months demonstrating a substantially reduced likelihood of developing autistic traits. Notably, this protective association exhibited a duration-dependent pattern, where an extended breastfeeding duration was correlated with progressively stronger neurodevelopmental benefits. Although few studies have investigated the dose–response relationship between breastfeeding duration and autistic traits, our result was consistent with some studies that have explored such a relationship between other childhood behavioral and emotional developments and breastfeeding duration [50]. Further studies are needed to explore and confirm how long breastfeeding reduces the risk of autism. Thus, our findings support the WHO recommendation for exclusive breastfeeding for the first 6 months of life and for breastfeeding children up to 2 y of age or beyond [19].

Our study revealed a gender difference. Breastfeeding duration was negatively associated with autistic traits in boys, but not in girls. This finding is significant, as gender’s moderating role is often overlooked in studies examining breastmilk’s impact on neurodevelopmental outcomes. Our results underscore the need to explore the mechanisms behind these gender differences. Previous research has shown that breastfeeding in early life influences neurodevelopment [52,53,54]. Gender differences in the impact of breastfeeding on children’s neurobehavioral development are influenced by a complex interplay of hormonal, genetic, nutritional, immunological, social, and cultural factors. Hormonal components in breastmilk, such as prolactin, oxytocin, and thyroid hormones, may affect male and female infants differently, with sex-specific sensitivity to hormonal regulation [55]. Genetic predisposition and epigenetic programming also play roles, shaping maternal lactation capacity, infant nutrient absorption, and potential interactions with breastmilk components [56]. Breastmilk contains essential fatty acids for brain development, which male infants may rely on more heavily, and its immunoglobulins and cytokines may provide greater protective benefits to males [57,58,59]. Social circumstances and cultural beliefs influence breastfeeding patterns [60,61], and maternal stress during lactation can alter milk composition [62], potentially affecting male neurodevelopment more significantly. Additionally, sex-sensitive developmental windows exist, with components in breastmilk exerting stronger effects on male neurodevelopment during periods of rapid brain growth [63,64]. Furthermore, boys may be more sensitive to breastmilk nutrients due to the “male disadvantage” [59]. However, the mechanisms driving these gender-specific effects of breastmilk on ASD remain unclear and warrant further investigation.

Several potential mechanisms may elucidate the link between breastfeeding practices and the manifestation of autistic traits in children. Breastmilk is replete with various substances, such as essential fatty acids, insulin-like growth factor, oxytocin, melatonin, and long-chain polyunsaturated fatty acids (LC-PUFAs), which are crucial for brain development and maturation [65,66,67,68,69,70,71,72]. Moreover, breastmilk contains immune factors that safeguard children against a multitude of infections. Studies have indicated that breastfed children are less prone to developing ear, throat, and sinus infections compared to those who are not breastfed [73,74]. Moreover, breastmilk contains immune factors that safeguard children against a multitude of infections. Studies have indicated that breastfed children are less prone to developing ear, throat, and sinus infections compared to those who are not breastfed [73]. Breastmilk also plays a pivotal role in the development of the gut microbiome, which may contribute to the etiology of neurodevelopmental disorders [75]. Research suggests that alterations in the gut microbiome may also contribute to the etiology of neurodevelopmental disorders, including ASD [76,77]. Physical and social interactions between the mother and child are alternative hypotheses that could explain the beneficial effects of breastfeeding. Further investigations are needed to understand how breastfeeding promotes neurodevelopment.

This study boasts several notable strengths, particularly the inclusion of a substantial sample of children exhibiting autistic traits, whose classifications were verified by their mothers using standardized instruments (ABC). Furthermore, the extensive data gathered through our questionnaires enabled adjustments for a multitude of maternal, child, and family characteristics, thereby facilitating a thorough assessment of the relationship between ASD and breastfeeding duration. However, several limitations of the present study should also be acknowledged. Firstly, the cross-sectional study design does not allow us to establish causality in the associations observed, and, thus, the results should be interpreted with caution and longitudinal studies are needed to establish a temporal relationship between breastfeeding and autistic traits. Secondly, the recruitment of all participants from Longhua District of Shenzhen may limit the generalizability of our findings to other populations. In addition, breastfeeding practices and child development in China are shaped by a multifaceted interplay of regional disparities and socio-economic factors. Urban areas, with better access to healthcare facilities, including Baby-Friendly Hospitals (BFHIs), generally have higher breastfeeding rates [78]. However, urban mothers often face workplace pressures, leading to shorter maternity leave and a reduced exclusive breastfeeding duration [79]. Conversely, rural areas, despite significant improvements in institutional delivery rates, still struggle with cultural practices and reliance on traditional advice, which can delay breastfeeding [80]. Additionally, geographic disparities in healthcare infrastructure and access to breastfeeding-friendly workplaces or community support pose challenges for migrant populations in cities [81]. These factors have a significant impact on child neurodevelopment. Therefore, further research is required to replicate our findings in diverse populations using prospective cohort studies that incorporate objective measures of the child behavioral problems assessed in this study, along with a broader range of potential covariates. Thirdly, the reliability of our results may be impacted by biases in the self-reported breastfeeding data due to factors like recall inaccuracies. Thirdly, our survey could not comprehensively assess all potential confounding factors, such as genetics and environmental influences (e.g., pollution and diet), known to influence neurodevelopment, which may impact the comprehensiveness and accuracy of our findings. Additionally, we did not collect data on maternal mental health, such as postpartum anxiety, which could potentially influence feeding practices and children’s behavioral outcomes. Moreover, we did not collect data on the psychiatric history of the parents, such as ASD and ADHD, which could potentially influence the associations between breastfeeding duration and the risk of ASD in children. Finally, we only used parent-reported screening tools to measure autistic traits in children, and did not use the DSM-V (The Diagnostic and Statistical Manual of Mental Disorders) to confirm ASD. Future studies need to use more tools to screen and diagnose mental disorders in children.

## 5. Conclusions

The findings of the present study suggest that both the duration of exclusive breastfeeding and the overall breastfeeding period in early life exert beneficial effects on the subsequent development of autistic traits in children. Moreover, there exist dose–response relationships between the duration of exclusive breastfeeding, the duration of breastfeeding, and the manifestation of autistic traits in children. This underscores the significance of adequate postnatal nutrition for infant neurodevelopment. In particular, our study reveals that exclusive breastfeeding during the first 2–6 months or an overall breastfeeding period exceeding 8 months can reduce the risk of childhood autistic traits. These results enhance our understanding of the health advantages of breastfeeding for children, thereby aiding in the formulation of breastfeeding guidelines aimed at improving global childhood health. This research is important to inform policy and clinical practice concerning nutritional recommendations for infants at a higher risk of developing ASD. However, it is necessary to conduct longitudinal studies across various regions and among diverse populations, with a particular emphasis on regular follow-ups with mothers. These studies are crucial for determining breastfeeding patterns and precise durations, which will help to validate our findings and provide deeper insights into the underlying mechanisms of these associations.

## Figures and Tables

**Table 1:** Characteristics of the study samples.

Characteristics	Overall (*n* = 17,382)	Children Without Autistic Traits (*n* = 16,716)	Children with Autistic Traits (*n* = 666)	*p*
Child gender (%)				<0.001
Boy	9413 (54.2)	8991 (53.8)	422 (63.4)	
Girl	7969 (45.8)	7725 (46.2)	244 (36.6)	
Child age (mean (SD))	3.48 (0.27)	3.48 (0.27)	3.46 (0.26)	0.080
Birth weight (mean (SD))	3419.37 (854.90)	3419.01 (851.91)	3428.42 (927.33)	0.781
Birth length (mean (SD))	51.01 (5.50)	51.04 (5.49)	50.24 (5.70)	<0.001
Maternal age at child’s birth (mean (SD))	29.18 (4.19)	29.22 (4.19)	28.13 (4.16)	<0.001
Paternal age at child’s birth (mean (SD))	34.75 (4.67)	34.78 (4.67)	33.82 (4.63)	<0.001
Maternal education level				<0.001
Junior high school or lower	4711 (27.1)	4458 (26.7)	253 (38.0)	
High school	7156 (41.2)	6911 (41.3)	245 (36.8)	
College or higher	5515 (31.7)	5347 (32.0)	168 (25.2)	
Paternal education level				<0.001
Junior high school or lower	4418 (25.4)	4184 (25.0)	234 (35.1)	
High school	6075 (34.9)	5858 (35.0)	217 (32.6)	
College or higher	6889 (39.6)	6674 (39.9)	215 (32.3)	
Family income (CNY/month)				<0.001
<5000	4003 (23.0)	3800 (22.7)	203 (30.5)	
5001–10,000	6249 (36.0)	5991 (35.8)	258 (38.7)	
10,001–20,000	3771 (21.7)	3643 (21.8)	128 (19.2)	
>20,000	3359 (19.3)	3282 (19.6)	77 (11.6)	
Marital status				0.003
Married	16,890 (97.2)	16,257 (97.3)	633 (95.0)	
Single	492 (2.8)	459 (2.7)	33 (5.0)	
Single child or not				0.004
No	9573 (55.1)	9243 (55.3)	330 (49.5)	
Yes	7809 (44.9)	7473 (44.7)	336 (50.5)	
Threatened abortion				0.011
No	15,022 (86.4)	14,469 (86.6)	553 (83.0)	
Yes	2360 (13.6)	2247 (13.4)	113 (17.0)	
Gestational hypertension				0.008
No	17,019 (97.9)	16,377 (98.0)	642 (96.4)	
Yes	363 (2.1)	339 (2.0)	24 (3.6)	
Preeclampsia/eclampsia				0.009
No	17,303 (99.5)	16,645 (99.6)	658 (98.8)	
Yes	79 (0.5)	71 (0.4)	8 (1.2)	
Gestational diabetes mellitus				0.540
No	16,142 (92.9)	15,528 (92.9)	614 (92.2)	
Yes	1240 (7.1)	1188 (7.1)	52 (7.8)	
Preterm birth				0.896
No	16,041 (92.3)	15,425 (92.3)	616 (92.5)	
Yes	1341 (7.7)	1291 (7.7)	50 (7.5)	
Time of adding complementary food (mean (SD))	5.98 (2.36)	5.98 (2.34)	5.92 (2.69)	0.537
The duration of exclusive breastfeeding (mean (SD))	3.34 (3.16)	3.35 (3.16)	3.02 (3.02)	0.007
Exclusive breastfeeding in the first 6 months of life				0.030
No	12,907 (74.3)	12,388 (74.1)	519 (77.9)	
Yes	4475 (25.7)	4328 (25.9)	147 (22.1)	
The duration of breastfeeding (mean (SD))	8.70 (5.72)	8.76 (5.75)	8.31 (5.57)	<0.001
Overall breastfeeding time				0.001
≤6 moths	7015 (39.3)	5951 (38.9)	1064 (41.6)	
7–12 moths	7375 (41.3)	6318 (41.3)	1057 (41.4)	
≥13 moths	3477 (19.5)	3043 (19.9)	434 (17.0)	

**Table 2:** Comparison of children’s autistic traits by their status of exclusive breastfeeding in the first 6 months of life.

Scales	Overall (*n* = 17,382)	Exclusive Breastfeeding in the First 6 Months of Life		*p*
		No (*n* = 12,907)	Yes (*n* = 4475)	
Autistic traits (%)				0.030
No	16,716 (96.20)	12,388 (96.00)	4328 (96.70)	
Yes	666 (3.80)	519 (4.00)	147 (3.30)	
ABC scores (mean (SD))	6.04 (11.82)	6.21 (12.12)	5.55 (10.88)	0.001
Sensory scores (mean (SD))	0.82 (2.33)	0.85 (2.38)	0.74 (2.18)	0.009
Relating scores (mean (SD))	1.98 (4.04)	2.02 (4.11)	1.88 (3.83)	0.046
Body and object use scores (mean (SD))	0.55 (2.00)	0.57 (2.04)	0.52 (1.87)	0.141
Language scores (mean (SD))	0.97 (2.79)	1.01 (2.86)	0.85 (2.58)	0.001
Social and self-help scores (mean (SD))	1.68 (3.09)	1.72 (3.15)	1.55 (2.91)	0.001

**Table 3:** Comparison of children’s autistic traits by their status of overall breastfeeding time.

Scales	Overall (*n* = 17,382)	Overall Breastfeeding Time			*p*	*p* for Trend
		≤6 Moths (*n* = 6731)	7–12 Moths (*n* = 7238)	≥13 Moths (*n* = 3413)		
Autistic traits (%)					0.001	<0.001
No	16,716 (96.20)	6435 (95.60)	6968 (96.30)	3313 (97.10)		
Yes	666 (3.80)	296 (4.40)	270 (3.70)	100 (2.90)		
ABC scores (mean (SD))	6.04 (11.82)	6.48 (12.68)	5.88 (11.67)	5.50 (10.25)	<0.001	<0.001
Sensory scores (mean (SD))	0.82 (2.33)	0.90 (2.51)	0.80 (2.29)	0.72 (2.01)	<0.001	<0.001
Relating scores (mean (SD))	1.98 (4.04)	2.08 (4.24)	1.94 (3.99)	1.88 (3.73)	0.034	<0.001
Body and object use scores (mean (SD))	0.55 (2.00)	0.61 (2.12)	0.54 (2.01)	0.46 (1.72)	0.001	<0.001
Language scores (mean (SD))	0.97 (2.79)	1.07 (3.00)	0.93 (2.74)	0.86 (2.47)	<0.001	<0.001
Social and self-help scores (mean (SD))	1.68 (3.09)	1.79 (3.22)	1.63 (3.04)	1.56 (2.90)	<0.001	<0.001

**Table 4:** Associations of exclusive breastfeeding in the first 6 months of life with children’s autistic traits ^†^.

Items	cOR (95%CI)	*p*	aOR (95%CI)	*p*
Autistic traits	0.811 (0.673, 0.977)	0.0273	0.852 (0.705, 1.030)	0.0981

^†^ Adjusted for child’s gender, birth weight (g), birth length (cm), preterm birth status, whether the child was an only child, parents’ age at child’s birth, parents’ marital status, parents’ education level, family income, supplementation time (month), threatened abortion, and gestational diseases (gestational hypertension and preeclampsia/eclampsia).

**Table 5:** Associations of exclusive breastfeeding in the first 6 months of life with children’s ABC scores ^†^.

Items	cβ (95%CI)	*p*	aβ (95%CI)	*p*
ABC scores	−0.653 (−1.054, −0.251)	0.0015	−0.528 (−0.932, −0.124)	0.0104
Sensory scores	−0.105 (−0.184, −0.026)	0.0093	−0.078 (−0.157, 0.002)	0.0562
Relating scores	−0.14 (−0.277, −0.003)	0.0459	−0.109 (−0.248, 0.029)	0.1225
Body and object use scores	−0.051 (−0.119, 0.017)	0.141	−0.042 (−0.111, 0.027)	0.2307
Language scores	−0.162 (−0.257, −0.067)	<0.001	−0.12 (−0.215, −0.025)	0.0137
Social and self-help scores	−0.176 (−0.281, −0.071)	0.001	−0.157 (−0.263, −0.052)	0.0035

^†^ Adjusted for child’s gender, birth weight (g), birth length (cm), preterm birth status, whether the child was an only child, parents’ age at child’s birth, parents’ marital status, parents’ education level, family income, supplementation time (month), threatened abortion, and gestational diseases (gestational hypertension and preeclampsia/eclampsia).

**Table 6:** Logistic regression table of the relationship between breastfeeding time and autistic traits in children ^†^.

Variable	cOR (95%CI)	*p*	aOR (95%CI)	*p*
Exclusive breastfeeding time				
<2 months	1.000 (Reference)		1.000 (Reference)	
≥2 months	0.815 (0.698, 0.952)	0.0099	0.848 (0.725, 0.993)	0.0404
Overall breastfeeding time				
<8 months	1.000 (Reference)		1.000 (Reference)	
≥8 months	0.775 (0.664, 0.905)	0.0013	0.798 (0.682, 0.934)	0.0050

^†^ Adjusted for child’s gender, birth weight (g), birth length (cm), preterm birth status, whether the child was an only child, parents’ age at child’s birth, parents’ marital status, parents’ education level, family income, supplementation time (month), threatened abortion, and gestational diseases (gestational hypertension and preeclampsia/eclampsia).

**Table 7:** Associations of overall breastfeeding time with children’s autistic traits ^†^.

Items	7–12 Months (*n* = 6318)				≥13 Months (*n* = 3043)			
	cOR (95%CI)	*p*	aOR (95%CI)	*p*	cOR (95%CI)	*p*	aOR (95%CI)	*p*
Autistic traits	0.842 (0.712, 0.997)	0.0459	0.844 (0.712, 1.001)	0.0508	0.656 (0.521, 0.826)	<0.001	0.709 (0.561, 0.897)	0.0041

^†^ Adjusted for child’s gender, birth weight (g), birth length (cm), preterm birth status, whether the child was an only child, parents’ age at child’s birth, parents’ marital status, parents’ education level, family income, supplementation time (month), threatened abortion, and gestational diseases (gestational hypertension and preeclampsia/eclampsia).

**Table 8:** Associations of overall breastfeeding time with children’s ABC scores ^†^.

Items	7–12 Months (*n* = 6318)				≥13 Months (*n* = 3043)			
	cβ (95%CI)	*p*	aβ (95%CI)	*p*	cβ (95%CI)	*p*	aβ (95%CI)	*p*
ABC scores	−0.608 (−1.000, −0.215)	0.0024	−0.618 (−1.009, −0.226)	0.0020	−0.979 (−1.465, −0.492)	<0.001	−0.724 (−1.213, −0.234)	0.0038
Sensory scores	−0.105 (−0.182, −0.027)	0.0080	−0.111 (−0.188, −0.033)	0.0050	−0.187 (−0.282, −0.091)	<0.001	−0.143 (−0.239, −0.046)	0.0038
Relating scores	−0.135 (−0.269, −0.001)	0.0476	−0.132 (−0.266, 0.003)	0.0546	−0.2 (−0.366, −0.033)	0.0187	−0.133 (−0.301, 0.035)	0.1211
Body and object use scores	−0.071 (−0.137, −0.004)	0.0374	−0.069 (−0.136, −0.003)	0.0409	−0.154 (−0.237, −0.072)	<0.001	−0.125 (−0.208, −0.042)	0.0032
Language scores	−0.147 (−0.240, −0.055)	0.0019	−0.147 (−0.239, −0.054)	0.0018	−0.217 (−0.332, −0.102)	<0.001	−0.151 (−0.267, −0.036)	0.0104
Social and self-help scores	−0.160 (−0.262, −0.057)	0.0022	−0.166 (−0.268, −0.063)	0.0015	−0.228 (−0.355, −0.100)	<0.001	−0.173 (−0.301, −0.045)	0.0083

^†^ Adjusted for child’s gender, birth weight (g), birth length (cm), preterm birth status, whether the child was an only child, parents’ age at child’s birth, parents’ marital status, parents’ education level, family income, supplementation time (month), threatened abortion, and gestational diseases (gestational hypertension and preeclampsia/eclampsia).

**Table 9:** Associations of exclusive breastfeeding duration and overall breastfeeding time with autistic traits in various subgroups among children ^†^*.

Characteristic	OR (95% CIs)			*p* Value for Interaction
Exclusive Breastfeeding in the First 6 Months of Life	No	Yes		
Gender				0.565
Boy	1 [Reference]	0.780 (0.620–0.990)		
Girl	1 [Reference]	0.880 (0.650–1.180)		
Maternal age at child’s birth (Years)				0.468
>35	1 [Reference]	1.060 (0.500–2.250)		
≤35	1 [Reference]	0.790 (0.650–0.960)		
Paternal age at child’s birth (Years)				0.805
>40	1 [Reference]	0.740 (0.380–1.440)		
≤40	1 [Reference]	0.810 (0.670–0.990)		
Maternal education level				0.964
Junior high school or lower	1 [Reference]	0.810 (0.590–1.120)		
High school	1 [Reference]	0.840 (0.620–1.140)		
College or higher	1 [Reference]	0.870 (0.610–1.230)		
Paternal education level				0.931
Junior high school or lower	1 [Reference]	0.880 (0.630–1.230)		
High school	1 [Reference]	0.800 (0.580–1.110)		
College or higher	1 [Reference]	0.850 (0.620–1.160)		
Family income (CNY/month)				0.870
<5000	1 [Reference]	0.870 (0.610–1.240)		
5001–10,000	1 [Reference]	0.770 (0.570–1.040)		
10,001–20,000	1 [Reference]	0.810 (0.540–1.230)		
>20,000	1 [Reference]	0.980 (0.590–1.630)		
Marital status				0.246
Married	1 [Reference]	0.840 (0.690–1.010)		
Single	1 [Reference]	0.370 (0.130–1.080)		
Single child or not				0.131
No	1 [Reference]	0.690 (0.520–0.910)		
Yes	1 [Reference]	0.920 (0.720–1.190)		
Threatened abortion				0.620
No	1 [Reference]	0.800 (0.650–0.980)		
Yes	1 [Reference]	0.910 (0.570–1.460)		
Gestational hypertension				0.815
No	1 [Reference]	0.810 (0.670–0.980)		
Yes	1 [Reference]	0.930 (0.310–2.820)		
Preeclampsia/eclampsia				0.952
No	1 [Reference]	0.820 (0.680–0.990)		
Yes	1 [Reference]	0.000 (0.000-Inf)		
Adding complementary food at six months				0.988
No	1 [Reference]	0.830 (0.610–1.120)		
Yes	1 [Reference]	0.830 (0.660–1.050)		
Overall breastfeeding time	≤6 moths	7–12 moths	≥13 moths	
Gender				0.293
Boy	1 [Reference]	0.760 (0.620–0.940)	0.620 (0.460–0.820)	
Girl	1 [Reference]	1.010 (0.760–1.330)	0.720 (0.490–1.070)	
Maternal age at child’s birth (Years)				0.517
>35	1 [Reference]	0.870 (0.420–1.830)	1.030 (0.460–2.290)	
≤35	1 [Reference]	0.840 (0.700–0.990)	0.640 (0.500–0.810)	
Paternal age at child’s birth (Years)				0.341
>40	1 [Reference]	0.870 (0.480–1.580)	1.040 (0.540–1.990)	
≤40	1 [Reference]	0.830 (0.700–0.990)	0.620 (0.490–0.800)	
Maternal education level				0.387
Junior high school or lower	1 [Reference]	0.750 (0.570–0.990)	0.750 (0.510–1.090)	
High school	1 [Reference]	1.010 (0.770–1.330)	0.640 (0.420–0.950)	
College or higher	1 [Reference]	0.750 (0.540–1.060)	0.630 (0.410–0.970)	
Paternal education level				0.256
Junior high school or lower	1 [Reference]	0.770 (0.580–1.020)	0.770 (0.510–1.150)	
High school	1 [Reference]	0.980 (0.730–1.320)	0.880 (0.590–1.300)	
College or higher	1 [Reference]	0.820 (0.610–1.100)	0.500 (0.340–0.760)	
Family income (CNY/month)				0.008
<5000	1 [Reference]	0.560 (0.410–0.760)	0.670 (0.440–1.010)	
5001–10,000	1 [Reference]	1.180 (0.900–1.550)	0.750 (0.510–1.110)	
10,001–20,000	1 [Reference]	0.760 (0.510–1.120)	0.760 (0.470–1.230)	
>20,000	1 [Reference]	0.820 (0.510–1.330)	0.370 (0.170–0.800)	
Marital status				0.578
Married	1 [Reference]	0.860 (0.720–1.020)	0.660 (0.520–0.830)	
Single	1 [Reference]	0.670 (0.290–1.530)	0.920 (0.330–2.570)	
Single child or not				0.364
No	1 [Reference]	0.760 (0.600–0.960)	0.670 (0.480–0.920)	
Yes	1 [Reference]	0.940 (0.740–1.200)	0.640 (0.460–0.880)	
Threatened abortion				0.852
No	1 [Reference]	0.830 (0.690–1.000)	0.650 (0.510–0.840)	
Yes	1 [Reference]	0.940 (0.620–1.410)	0.650 (0.380–1.140)	
Gestational hypertension				0.049
No	1 [Reference]	0.870 (0.730–1.030)	0.690 (0.550–0.870)	
Yes	1 [Reference]	0.390 (0.150–1.020)	0.120 (0.020–0.890)	
Preeclampsia/eclampsia				0.211
No	1 [Reference]	0.850 (0.720–1.010)	0.670 (0.530–0.840)	
Yes	1 [Reference]	0.370 (0.070–1.980)	0.000 (0.000-Inf)	
Adding complementary food at six months				0.913
No	1 [Reference]	0.890 (0.680–1.150)	0.670 (0.450–1.000)	
Yes	1 [Reference]	0.830 (0.670–1.030)	0.680 (0.510–0.900)	

^†^ Adjusted for child’s gender, birth weight (g), birth length (cm), preterm birth status, whether the child was an only child, parents’ age at child’s birth, parents’ marital status, parents’ education level, family income, supplementation time (month), threatened abortion, and gestational diseases (gestational hypertension and preeclampsia/eclampsia). * The model is not adjusted for the stratification variable in each case.

## Data Availability

The data that support the findings of this study are available on request from the corresponding authors. The data are not publicly available due to privacy or ethical restrictions.

## Supplementary Materials

The following supporting information can be downloaded at: https://www.mdpi.com/article/10.3390/nu17050836/s1, Table S1: Risks of children’s autistic traits based on feeding pattern (exclusive breastfeeding vs. non- exclusive breastfeeding) for first 6 months. Table S2: Risks of children’s ABC scores based on feeding pattern (exclusive breastfeeding vs. non- exclusive breastfeeding) in the first 6 months stratified by children’s sex. Table S3: Protective Effect of Months of Exclusive Breastfeeding on Children’s ABC Scores Stratified by Sex. Table S4: Risks of children’s autistic traits based on overall breastfeeding time stratified by children’s sex. Table S5: Risks of children’s ABC scores based on overall breastfeeding time stratified by children’s sex. Table S6: Protective Effect of Breastfeeding on Children’s ABC Scores Stratified by Sex.
