# Supplementary material for: Impact of Breastfeeding Practices on Autistic Traits in Chinese Children Aged from 3 to 4 Years: Cross-Sectional Study
Source: Nutrients. 2025 Feb 28;17(5):836. doi: 10.3390/nu17050836 (PMC11901610; doi:10.3390/nu17050836)
Supplement: Supplementary file 1 [file nutrients-17-00836-s001.zip › nutrients-3504351-supplementary.pdf]

Table S1 Risks of children's autistic traits based on feeding pattern (exclusive breastfeeding vs. non- exclusive breastfeeding) for first 6 months stratified by children's sex*†.

Items	Boys (9413)			Girls (7969)		
	exclusive breastfeeding (n=2353)			exclusive breastfeeding (n=2122)		
	Events	aOR (95%CI)	*P*	Events	aOR (95%CI)	*P*
**Autistic traits#**	88 (20.9%)	0.906 (0.67, 1.225)	0.5216	59 (24.2%)	0.828 (0.649, 1.057)	0.1294

* The reference group was children who were not exclusively breastfed for first 6 months.

†Adjusted for birth weight (g), birth length (cm), preterm birth status, whether the child was an only child, parents' marital status, education level, family income, parity, parental age at the time of the child's birth, supplementation time (month), threatened abortion, and gestational diseases (gestational hypertension, preeclampsia/eclampsia).

Table S2 Risks of children's ABC scores based on feeding pattern (exclusive breastfeeding vs. non- exclusive breastfeeding) in the first 6 months stratified by children's sex*†.

Items	Boys (9413)		Girls (7969)	
	aβ (95%CI)	*P*	aβ (95%CI)	*P*
**ABC scores**	**-0.724 (-1.275, -0.173)**	**0.0100**	**-0.370 (-0.954, 0.213)**	**0.2134**
**Sensory scores**	-0.156 (-0.268, -0.043)	0.0067	-0.012 (-0.124, 0.100)	0.8293
**Relating scores**	-0.198 (-0.392, -0.004)	0.0451	-0.034 (-0.231, 0.162)	0.7322
**Body and object use scores**	-0.067 (-0.164, 0.030)	0.1737	-0.022 (-0.119, 0.074)	0.6516
**Language scores**	-0.087 (-0.210, 0.035)	0.1613	-0.151 (-0.294, -0.008)	0.0386
**Social and self-help scores**	-0.196 (-0.340, -0.053)	0.0073	-0.125 (-0.278, 0.028)	0.1105

* The reference group was children who were not breastfed for first 6 months.

† Adjusted for birth weight (g), birth length (cm), preterm birth status, whether the child was an only child, parents' marital status, education level, family income, parity, parental age at the time of the child's birth, supplementation time (month), threatened abortion, and gestational diseases (gestational hypertension, preeclampsia/eclampsia).

Table S3 Protective Effect of Months of Exclusive Breastfeeding on Children's ABC Scores Stratified by Sex[*†].

Items	Boys (9413)		Girls (7969)	
	aβ (95%CI)	*P*	aβ (95%CI)	*P*
**ABC scores**	**-0.102 (-0.178, -0.027)**	**0.0080**	**-0.075 (-0.156, 0.006)**	**0.0689**
**Sensory scores**	-0.027 (-0.043, -0.012)	< 0.001	-0.006 (-0.021, 0.010)	0.4876
**Relating scores**	-0.031 (-0.058, -0.005)	0.0213	-0.017 (-0.044, 0.011)	0.2334
**Body and object use scores**	-0.007 (-0.021, 0.006)	0.2839	-0.003 (-0.017, 0.010)	0.6244
**Language scores**	-0.012 (-0.029, 0.005)	0.1628	-0.023 (-0.043, -0.003)	0.0221
**Social and self-help scores**	-0.022 (-0.042, -0.002)	0.0291	-0.024 (-0.045, -0.002)	0.0299

[†]Adjusted for birth weight (g), birth length (cm), preterm birth status, whether the child was an only child, parents' marital status, education level, family income, parity, parental age at the time of the child's birth, supplementation time (month), threatened abortion, and gestational diseases (gestational hypertension, preeclampsia/eclampsia).

Table S4 Risks of children's autistic traits based on overall breastfeeding time stratified by children's sex†.

Items	Boys (9413)						Girls (7969)					
	7-12 months (n=3874)			≥13 months (n = 1882)			7-12 months (n=3364)			≥13 months (n = 1531)		
	Events	aOR (95%CI)	*P*	Events	aOR (95%CI)	*P*	Events	aOR (95%CI)	*P*	Events	aOR (95%CI)	*P*
**Autistic traits**	**161 (38.2%)**	**0.770 (0.621, 0.955)**	**0.0176**	**64 (15.2%)**	**0.690 (0.515, 0.925)**	**0.0129**	**109 (44.7%)**	**0.994 (0.751, 1.316)**	**0.9667**	**36 (14.8%)**	**0.754 (0.509, 1.118)**	**0.1598**

†Adjusted for birth weight (g), birth length (cm), preterm birth status, whether the child was an only child, parents' marital status, education level, family income, parity, parental age at the time of the child's birth, supplementation time (month), threatened abortion, and gestational diseases (gestational hypertension, preeclampsia/eclampsia).

Table S5 Risks of children's ABC scores based on overall breastfeeding time stratified by children's sex†.

Items	Boys (9413)				Girls (7969)			
	7-12 months (n=3874)		≥13 months (n = 1882)		7-12 months (n=3364)		≥13 months (n = 1531)	
	aβ (95%CI)	*P*	aβ (95%CI)	*P*	aβ (95%CI)	*P*	aβ (95%CI)	*P*
**ABC scores**	**-0.798 (-1.359, -0.237)**	**0.0053**	**-0.885 (-1.580, -0.189)**	**0.0127**	**-0.401 (-0.940, 0.137)**	**0.1443**	**-0.500 (-1.181, 0.181)**	**0.1498**
**Sensory scores**	-0.134 (-0.242, -0.026)	0.0150	-0.186 (-0.320, -0.053)	0.0063	-0.086 (-0.196, 0.024)	0.1257	-0.085 (-0.224, 0.055)	0.2339
**Relating scores**	-0.182 (-0.371, 0.007)	0.0593	-0.15 (-0.384, 0.084)	0.2099	-0.068 (-0.257, 0.122)	0.4845	-0.102 (-0.341, 0.138)	0.4048
**Body and object use scores**	-0.046 (-0.139, 0.047)	0.3328	-0.124 (-0.239, -0.009)	0.0345	-0.101 (-0.196, -0.006)	0.0369	-0.125 (-0.245, -0.005)	0.0407
**Language scores**	-0.203 (-0.340, -0.065)	0.0039	-0.240 (-0.410, -0.069)	0.0059	-0.0780 (-0.198, 0.041)	0.1998	-0.038 (-0.189, 0.113)	0.6245
**Social and self-help scores**	-0.238 (-0.385, -0.090)	0.0016	-0.181 (-0.363, 0.002)	0.0525	-0.078 (-0.218, 0.062)	0.2744	-0.157 (-0.334, 0.020)	0.0824

†Adjusted for birth weight (g), birth length (cm), preterm birth status, whether the child was an only child, parents' marital status, education level, family income, parity, parental age at the time of the child's birth, supplementation time (month), threatened abortion, and gestational diseases (gestational hypertension, preeclampsia/eclampsia).

Table S6 Protective Effect of Breastfeeding on Children's ABC Scores Stratified by Sex †.

Items	Boys (9413)		Girls (7969)	
	**aβ (95%CI)**	***P***	**aβ (95%CI)**	***P***
**ABC scores**	**-0.072 (-0.117, -0.028)**	**0.0015**	**-0.060 (-0.103, -0.016)**	**0.0075**
**Sensory scores**	-0.013 (-0.022, -0.005)	0.0021	-0.011 (-0.020, -0.002)	0.0123
**Relating scores**	-0.017 (-0.032, -0.002)	0.0299	-0.015 (-0.031, 0)	0.0520
**Body and object use scores**	-0.008 (-0.015, -0.001)	0.0320	-0.010 (-0.018, -0.002)	0.0104
**Language scores**	-0.017 (-0.028, -0.007)	0.0018	-0.008 (-0.018, 0.001)	0.0960
**Social and self-help scores**	-0.016 (-0.028, -0.005)	0.0058	-0.015 (-0.026, -0.004)	0.0098

†Adjusted for birth weight (g), birth length (cm), preterm birth status, whether the child was an only child, parents' marital status, education level, family income, parity, parental age at the time of the child's birth, supplementation time (month), threatened abortion, and gestational diseases (gestational hypertension, preeclampsia/eclampsia).
